# Use of Branched-Chain Amino Acids as a Potential Treatment for Improving Nutrition-Related Outcomes in Advanced Chronic Liver Disease

**DOI:** 10.3390/nu15194190

**Published:** 2023-09-28

**Authors:** Santo Colosimo, Simona Bertoli, Francesca Saffioti

**Affiliations:** 1 School of Nutrition Science, University of Milan, 20133 Milan, Italy; 2 Department of Food, Environmental and Nutritional Sciences (DeFENS), University of Milan, 20133 Milan, Italy; 3 Laboratory of Clinical Studies on Obesity, Istituto Auxologico Italiano IRCCS, 20145 Milan, Italy; 4 Oxford Liver Unit, Department of Gastroenterology and Hepatology, Oxford University Hospitals NHS Foundation Trust, Oxford OX3 9DU, UK; francesca.saffioti@ouh.nhs.uk

**Keywords:** ACLD, sarcopenia, hepatic encephalopathy, BCAAs

## Abstract

Advanced chronic liver disease (ACLD) represents a complex and multifactorial clinical entity characterized by liver dysfunction and associated complications. In recent years, the significance of nutritional status in ACLD prognosis has gained considerable attention. This review article delves into the multifactorial pathogenesis of malnutrition in ACLD and its profound consequences for health outcomes. We explore the clinical implications of secondary sarcopenia in ACLD and highlight the critical relevance of frailty in both decompensated and compensated ACLD. A specific focus of this review revolves around branched-chain amino acids (BCAAs) and their pivotal role in managing liver disease. We dissect the intricate relationship between low Fischer’s ratio and BCAA metabolism in ACLD, shedding light on the molecular mechanisms involved. Furthermore, we critically evaluate the existing evidence regarding the effects of BCAA supplementation on outcomes in ACLD patients, examining their potential to ameliorate the nutritional deficiencies and associated complications in this population.

## 1. Introduction

In recent years, the relationship between nutritional status and clinical outcomes in patients with advanced chronic liver disease (ACLD) has become increasingly clear with the increasing body of evidence published in the medical literature. ACLD refers to the advanced stage of liver disease characterized by structural changes of the liver parenchyma (histological evidence of liver fibrosis, regenerative nodules, and cirrhosis) and impaired liver function [1]. Malnutrition has been demonstrated to be associated with mortality, the development of complications, reduced survival, and reduced health-related quality of life (HRQoL) in individuals with ACLD [2,3,4].

Studies investigating the use of branched-chain amino acids (BCAAs), a group of essential amino acids, namely valine, leucine, and isoleucine, as a treatment for malnutrition in ACLD in adults provide contrasting findings. As such, the use of BCAA supplementation in clinical practice remains controversial, in particular with regards to the optimal dose, duration, and relative concentration of amino acids, as there was significant variability across the studies. The current international guidelines for the treatment of hepatic encephalopathy (HE) [5,6], one of the most serious complications of ACLD, suggest that BCAAs have a beneficial effect on HE and can be used as second-line therapy in patients with HE not responding to conventional therapy.

However, the question as to whether the use of BCAAs is of benefit in the treatment of malnutrition in ACLD is not entirely addressed. This review aims to critically evaluate the current evidence on the efficacy of BCAA supplementation in adults with ACLD, with a focus on changes in anthropometric measurements, body composition, muscle strength, liver biomarkers, medical and hepatic complications (HE, ascites, oedema), and patient-centered outcomes (event-free survival and health-related quality of life).

## 2. The Role of Nutritional Status in the Prognosis of Advanced Chronic Liver Disease

### 2.1. The Multifactorial Pathogenesis of Malnutrition in ACLD and Its Consequences on Health Outcomes

Advanced chronic liver disease (ACLD) [1] typically occurs as a consequence of chronic liver injury from various causes, including chronic viral hepatitis, alcohol excess, metabolic (non-alcoholic) fatty liver disease, autoimmune and cholestatic liver diseases, and other less frequent etiologies. It represents a significant global health burden due to its association with high morbidity and mortality rates [7]. Patients with ACLD often experience a deteriorated quality of life and malnutrition, a well-known poor prognostic factor affecting up to 70–75% of both adults and children with chronic liver disease [4,8,9,10]. In patients with ACLD, malnutrition is associated with the progression of liver failure, an increased occurrence of complications such as ascites, hepatic encephalopathy, and infections, and a reduced survival rate [2,3,4]. The etiology of malnutrition in ACLD is multifactorial and related to poor nutrient intake, altered nutrient absorption and utilization, and increased resting energy expenditure [4]. The prevalence of malnutrition is associated with the severity of liver impairment to the extent that in individuals with ACLD in the Child–Pugh C stage, the percentage of malnutrition reaches 95% [6,11].

One of the most worrisome complications of chronic liver disease is hepatocellular carcinoma (HCC), which has an incidence of about 1.5% per year and is currently the third most common cause of cancer-related mortality worldwide [12]. Recent data suggest that impaired nutritional status is a frequent issue in patients with HCC and likely subsequent, at least in part, to the underlying cirrhosis. This impaired nutritional status is causally related to impaired overall and complication-free survival [13]. The identification and accurate staging of ACLD are crucial for effective management and appropriate nutritional interventions.

### 2.2. The Importance of Addressing Secondary Sarcopenia in ACLD

Sarcopenia, a condition characterized by generalized loss of skeletal muscle volume and strength, can be classified as primary, due to aging, or secondary, due to underlying diseases such as chronic kidney and inflammatory diseases, malignant tumors, and ACLD [14,15]. The prevalence of sarcopenia in cirrhotic patients can vary greatly, from 30% to 70%, depending on the diagnostic methods used, the cut-offs for those methods, the patient’s phenotype, the degree of liver impairment, and whether or not the patient has hepatocellular carcinoma (HCC) [16]. The prevalence of secondary sarcopenia is high in patients with advanced fibrosis [3,9,15,17,18,19] and can worsen patient prognoses in ACLD [20]. Hence, early diagnosis and appropriate treatment are important. Sarcopenia has been shown to be a negative prognostic factor in liver transplant candidates. The effect of sarcopenia on the outcomes of patients undergoing transjugular porto-systemic shunts (TIPSS) is still controversial [21,22].

Strategies for treating sarcopenia related to chronic liver disease include exercise regimens and meals rich in calories and proteins, with supplementation with BCAAs [20,23,24].

### 2.3. Clinical Significance of Frailty in Decompensated and Compensated ACLD

Frailty is a complex syndrome characterized by a decline in physiological reserves and an increased susceptibility to health stressors [25]. In individuals with cirrhosis, frailty is commonly described as a reduction in physical function, diminished physical performance, and disability [26,27]. The reported prevalence of frailty in patients with ACLD ranges from 18% to 43% [28,29]. The clinical impact of frailty in individuals with decompensated ACLD has been shown to be associated with worse outcomes, such as waitlist mortality, hospitalization, and further decompensations [30,31,32]. A study aimed at assessing the accuracy of a frailty score in compensated ACLD has demonstrated a significant association between frailty and the development of new decompensation and unplanned hospitalization [33]. Additionally, frailty is linked to an increased risk of falls, depression, disability, and impaired quality of life [28,33], while an improvement in frailty scores over time has been shown to be associated with better clinical outcomes [34]. Consequently, there is an urgent need for therapeutic interventions that target the reversal of frailty in ACLD.

## 3. Branched-Chain Amino Acids (BCAAs) in Liver Disease

### 3.1. Low Fischer’s Ratio and BCAAs Metabolism in ACLD

A common characteristic observed in adults and children with ACLD is a decrease in serum BCAA concentration and an increase in aromatic amino acids (AAA), namely phenylalanine, tyrosine, and tryptophan [35], resulting in a low Fischer’s ratio (BCAA/AAA). The underlying cause of the low Fischer’s ratio in individuals with advanced liver disease has been attributed to increased oxidation and reduced endogenous disposal of BCAAs in skeletal muscle, along with alterations in AAA oxidation, indicating a higher BCAA requirement in chronic liver disease patients [36]. The metabolic changes in amino acid metabolism serve as a hallmark of liver disease and may become more pronounced with the progression of liver disease severity and the degree of malnutrition [37,38]. A low Fischer’s ratio has been found to be associated with the development of complications related to cirrhosis, such as hepatic encephalopathy [35].

**Figure 1:**
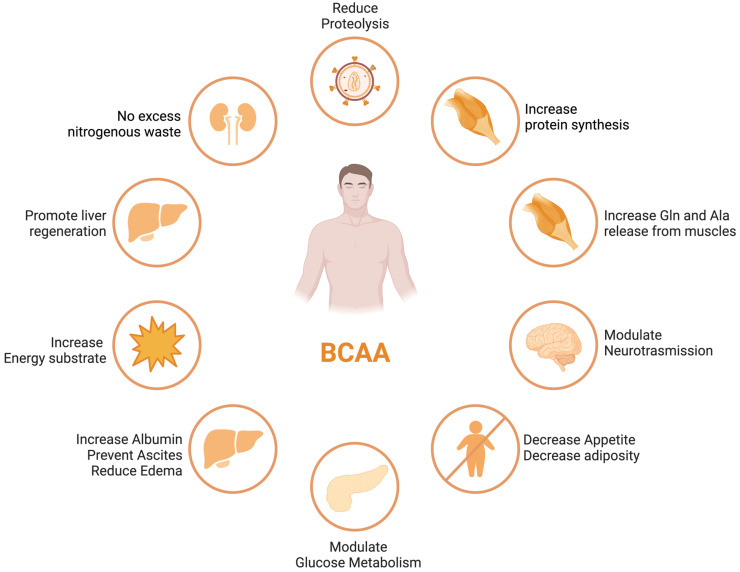
BCAA effects in different organs and tissues.

BCAA derived from the diet largely bypasses first-pass hepatic catabolism, and their catabolic disposal primarily occurs in skeletal muscle, making them a preferred source of amino acids in patients with liver disease [39]. Numerous clinical trials have demonstrated the beneficial effects of BCAA supplementation in liver failure, including improvements in nutritional status, HE, and health-related quality of life (HRQoL) [40,41] (see Figure 1). Nonetheless, these results were not replicated in other studies [42], leaving a degree of uncertainty with regards to the potential therapeutic efficacy of the different outcomes of ACLD.

ACLD patients frequently exhibit decreased serum BCAA concentrations, which is associated with a poor prognosis [43,44]. The correlation between decreased levels of circulating BCAAs and the extent of liver disease severity, along with compromised muscle function, indicates the potential utility of BCAAs as a valuable prognostic indicator for gauging the progression of liver disease [45].

**Figure 2:**
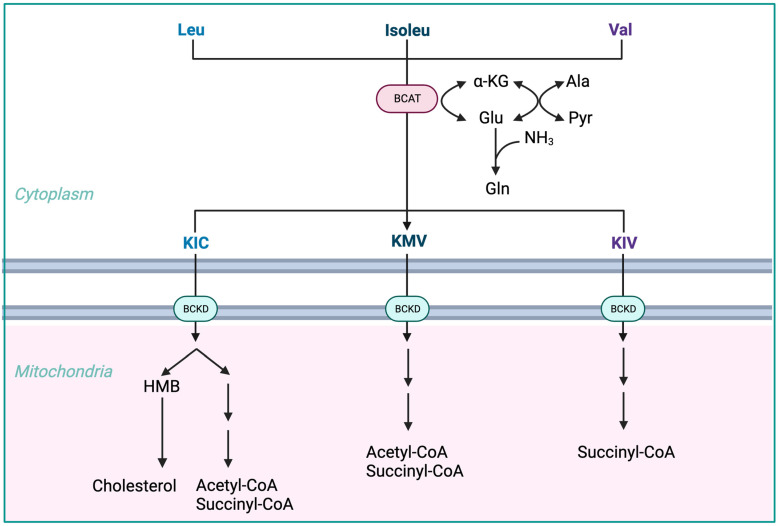
Advantages of BCAA-based nutritional formulas due to muscle dominance in BCAA catabolism. ALA—alanine; GLU—glutamate; GLN—glutamine; HMB—β-hydroxy-β-methylbutyrate; HMG-CoA—3-hydroxy-3-methylglutaryl-CoA; KIC—α-ketoisocaproate (ketoleucine); KIV—α-ketoisovalerate (ketovaline); KMV—α-keto-β-methylvalerate (ketoisoleucine); α-KG—α-ketoglutarate; BCAT—branched-chain-amino-acid aminotransferase; BCKD—branched-chain α-keto acid dehydrogenase. Modified from Holeček [51].

**Figure 3:**
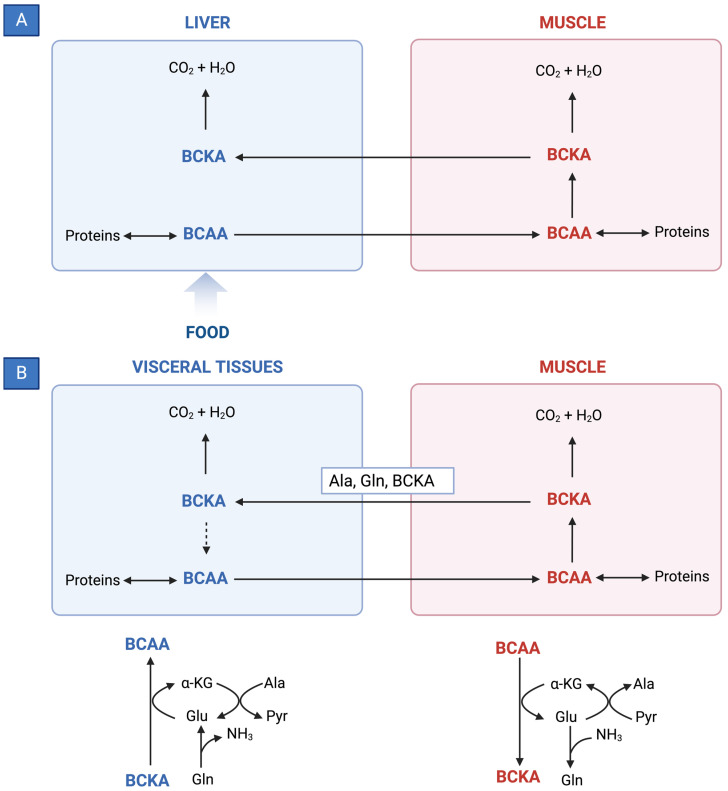
(**A**) Reversible BCAT reaction and amination process in tissue-specific BCAA metabolism. (**B**) Interorgan cycle: a mechanism to preserve BCAAs in physiological and pathological conditions. ALA—alanine; BCAAs—branched-chain amino acids; BCKAs—branched-chain keto acids; GLU—glutamate; GLN—glutamine; PYR—pyruvate; α-KG—α-ketoglutarate. Modified from Holeček [51].

BCAAs have a stimulatory effect on protein synthesis and/or an inhibitory effect on proteolysis [43], and BCAA supplementation leads to an increase in the serum concentration of BCAAs in cirrhotic patients [46,47,48] (Figure 1). Among the BCAAs, leucine plays a particularly crucial role in enhancing muscle protein synthesis [49]. All three BCAAs share similar metabolic pathways [50] and have been shown to exhibit antagonism in their respective catabolism at different intake levels, emphasizing the importance of prescribing optimal concentrations of each BCAA to maximize their benefits [50] (Figure 2 and Figure 3A,B).

- The initial stage of BCAA breakdown occurs outside the liver due to low BCAT activity, allowing BCAAs to quickly accumulate in the bloodstream, providing a unique advantage to BCAA-based nutritional formulas;
- Skeletal muscle plays a vital role in BCAA breakdown, facilitated by branched-chain amino acid aminotransferase (BCAT). This leads to the production of branched-chain keto acids (BCKAs), glutamate, alanine, and glutamine, which are released into the bloodstream;
- BCKD, an enzyme complex located in the inner mitochondrial membrane, irreversibly decarboxylates BCKAs into branched-chain acyl-CoA esters;
- The activity of BCKD is regulated through phosphorylation and dephosphorylation processes mediated by specific kinases and phosphatases;
- The liver exhibits the highest BCKD activity, while muscles, adipose tissue, and the brain have relatively lower activity levels;
- Muscles, comprising a significant proportion of total body weight, are major contributors to overall BCAA utilization, alongside the liver;
- Factors like cytokines, hormones, nutrients, and metabolites affect BCKD activity, with endotoxin or TNF-α administration inducing increased BCKD activity in muscles;
- BCAAs follow diverse metabolic pathways post-BCKD reaction, with KIC being ketogenic, KIV being glucogenic, and KMV being both glycogenic and ketogenic;
- The catabolism of KIC results in the synthesis of β-hydroxy-β-methylbutyrate (HMB) through the action of KIC dioxygenase.

### 3.2. Effects of BCAA Supplementation on Outcomes in ACLD

Recent nutritional guidelines from the European Association for the Study of Liver (EASL) [6] and the European Society of Parenteral and Enteral Nutrition (ESPEN) [52] recommend BCAA supplementation in subgroups of patients with liver cirrhosis, especially those with hepatic encephalothy (HE). The use of BCAAs is not exempt from mild adverse events. Potential adverse effects encompass esophageal reflux, enhanced insulin resistance, and disruptions in the sleep cycle.

#### 3.2.1. Hepatic Encephalopathy

Hepatic encephalopathy (HE) refers to the spectrum of potentially reversible neuropsychiatric abnormalities seen in patients with advanced, acute, or chronic liver disease, secondary to hepatic dysfunction, portosystemic shunting, or both. It manifests as a wide spectrum of neuropsychiatric abnormalities, from subclinical changes (mild cognitive impairment) to marked disorientation, confusion, and coma.

The exact pathophysiological mechanism is not known, although it is increasingly recognized that hyperammonemia, systemic inflammation, intestinal dysbiosis, and portal-systemic shunting, leading to an increased flow of neurotoxins across the blood-brain barrier, act in a synergistic manner, leading to the development of HE [53].

This condition can significantly impact a patient’s quality of life and is associated with poor survival and a high risk of recurrence [2,5,54].

There is evidence that, in particular, oral BCAA-enriched formulations improve the manifestations of episodic HE (both overt or minimal) [7,55] while intravenous supplementation does not appear to have any beneficial effect [56].

A Cochrane systematic review including 16 randomized clinical trials compared BCAAs to placebo, diet, lactulose, or neomycin in people with cirrhosis. The results showed that BCAAs had a beneficial effect on manifestations of HE, including overt hepatic encephalopathy (RR = 0.73, 95% CI 0.61–0.88) but not minimal hepatic encephalopathy. There were no beneficial or detrimental effects of BCAAs on mortality, quality of life, or nutritional outcomes [57].

A recent high-quality randomized clinical trial showed BCAAs did not prevent recurrence in patients with a previous episode of overt HE [35].

#### 3.2.2. Overall Survival

A recent meta-analysis of six studies was conducted to assess the effect of BCAA supplementation on overall survival in cirrhotic patients [13]. The studies included a total of 1253 patients who were followed for at least 6 months. The results showed that BCAA supplementation was not statistically significantly associated with an improvement in overall survival (RR = 0.58, 95% CI 0.34–1.00). However, one study found a statistically significant beneficial effect of BCAA supplementation on overall survival during hospital admission (mortality 12% vs. 47% in the supplemented and non-supplemented groups, respectively) [58]. The other studies did not find an effect of BCAA supplementation on overall survival [59,60,61].

As previously mentioned, no benefit to survival was found in Gluud’s Cochrane systematic review [57].

#### 3.2.3. Event-Free Survival

In the same meta-analysis of six studies, van Dijk et al. reviewed the effect of BCAA supplementation on event-free survival in ACLD patients [13]. The studies included a total of 1035 patients who were followed for at least 6 months (follow-up period from 1 to 3 years). The dosage of BCAA supplementation was approximately 12 g per day, and the tracked cirrhosis-related events included death, liver decompensation, and hepatocellular carcinoma. The results showed that BCAA supplementation was associated with a statistically significant improvement in event-free survival (RR = 0.61, 95% CI 0.42–0.88). As such, patients who received BCAA supplementation were 61% less likely to experience an event (such as hospitalization, liver decompensation, or death) than patients who did not receive BCAA supplementation. Four of the studies included in the meta-analysis were randomized controlled trials [4,62,63,64], one was a prospective study [65], and one study had a retrospective design [65]. When only the randomized controlled trials were included in the meta-analysis, a significant effect on event-free survival was still present (RR = 0.70, 95% CI 0.54–0.91).

#### 3.2.4. Nutritional Status

The same systematic review gathered eight available studies on BCAA supplementation and nutritional parameter outcomes [13]. The results of these studies are contradictory, as some show beneficial effects of BCAA supplementation and others show no effect or even harmful effects. A recent placebo-controlled trial on BCAAs in cirrhotic patients with sarcopenia found that BCAA supplementation had a beneficial effect on skeletal muscle index measured with a CT scan, but this study was small and did not correct for other factors that could have influenced the results [61].

#### 3.2.5. Quality of Life

Three out of the five studies reported in van Dijk’s systematic review found that BCAA supplementation improved one or more subscales of the SF-36, a health-related quality of life questionnaire [4,63,66]. No effect was reported in the remaining two studies [3,67]. The studies included a total of 875 patients, with 442 receiving BCAA supplementation and 433 receiving a placebo [13].

#### 3.2.6. Liver Disease Severity and Hepatocellular Carcinoma

Limited or conflicting evidence is available for the effectiveness of BCAA supplementation in most subgroups of chronic liver disease following specific therapeutic interventions for hepatocellular carcinoma (HCC) [68].

Van Dijk and colleagues conducted a systematic evaluation to assess the potential effects of BCAA supplementation in patients with chronic liver disease and coexisting HCC [68]. They examined various clinical circumstances, including general chronic liver disease as well as specific treatment situations such as hepatic resection, radiological intervention, systemic therapy for HCC, liver transplantation, large volume paracentesis for ascites, and endoscopic therapy of esophageal varices. The studies included a total of 1252 patients, with 581 patients receiving BCAA supplementation and 671 patients receiving placebo. The follow-up period of the studies varied between 1 and 3 years. No significant difference was found in the occurrence rates of HCC between the BCAAs and placebo groups (RR = 0.82, 95% CI 0.60–1.12). The exception was systemic therapy (sorafenib), where the two available retrospective studies reported better outcomes in patients receiving long-term BCAA supplementation compared to the control group [62,63].

The analysis focused on the effects of BCAA supplementation on various parameters reflecting liver function (Child–Pugh and MELD scores, serum albumin, clotting, and bilirubin concentrations) and revealed contradictory results with no overall significant effect.

Similarly, the effects of BCAA supplementation on sarcopenia showed inconsistent findings across different assessment methods (bioelectrical impedance analysis [7], mid-arm muscle circumference [9], and CT scan to measure skeletal muscle index [50]). No beneficial effects of BCAA supplementation on sarcopenia were reported in patients who underwent specific therapeutic interventions for HCC, such as resection, trans-arterial chemoembolization (TACE), or radiotherapy [68].

## 4. BCAA Supplementation and Body Composition: Evidence for Optimal Dose, Timing, and Formulations

In clinical trials involving adults, the primary method of delivering BCAAs is through oral administration, as supported by previous research [11,60]. The collective findings derived from comprehensive systematic reviews and meta-analyses reveal a broad spectrum of BCAA dosages, spanning from 5.5 to 30 g per day (g/day), or equivalently, 0.104 to 0.29 g per kilogram of body weight (g/kg) [60].

Significant heterogeneity is observed in the distribution of individual BCAAs among study participants. Specifically, isoleucine content ranged from 23% to 35%, leucine from 36% to 50%, and valine from 25% to 29%. Data pertaining to relative BCAA proportions were documented differently in the 24 studies conducted on adults. Among these, 10 studies presented both g/day and g/kg values [2,20,47,69,70,71,72,73,74,75], 12 studies exclusively measured BCAAs in g/day units [3,4,35,41,62,76,77,78,79,80,81,82], and 2 studies solely provided BCAA measurements in g/kg units [40,83]. Notably, only 6 of the adult trials aligned with the BCAA ratios found in egg protein, encompassing total doses ranging between 5.5 and 11 g/day, or 0.104 to 0.25 g/kg [41,67,73,76,80,83].

For adult subjects, BCAA supplementation was typically administered either once daily or fractionated across 2 to 4 meals. Frequently, such administration occurred during the late evening or through a combination of nocturnal and daytime intake. In the context of pediatric investigations, a solitary study reported BCAA dosages (0.33 to 0.39 g/kg) and associated proportions (isoleucine 24%, leucine 41%, and valine 35%), mirroring the composition found in egg protein.

Overall, the administration of BCAA supplements demonstrated a favorable safety profile across the board. In the majority of cases, any adverse effects were minimal and predominantly encompassed minor gastrointestinal discomfort, such as nausea or lack of palatability, as observed and reported in several studies [60].

Several studies investigated the effects of BCAAs on weight, fat mass, surrogate markers of muscle mass, and muscle strength in subjects with ACLD. A systematic review published in 2018 evaluated 40 studies from 1989 to 2017 and suggested that BCAA supplementation could potentially yield beneficial impacts on weight, fat mass, lean body mass, and serum albumin levels in patients with ACLD [84]. Although most studies included (76%) showed no significant effect on weight, and most studies (75%) reported minimal to no effects on fat mass and muscle mass after BCAAs administration, a pooled analysis showed a positive albeit small effect on muscle strength measured by handgrip, with an average 4% increase in the treatment group compared to a 6.8% reduction in the control group [84]. Baseline nutritional status was assessed in 29 studies on adults with cirrhosis. Patients with normal nutritional status at baseline exhibited better effects in response to BCAA supplementation compared to malnourished individuals, with regards to improvement of albumin levels (*n* = 17), muscle strength (*n* = 4), and HE (*n* = 4) [84].

## 5. Discussion and Conclusions

The use of BCAAs as therapeutic agents in ACLD is a promising area of research. Low plasma levels of BCAAs are associated with the development of cirrhosis complications such as sarcopenia and HE. Increasing data suggests that BCAA supplementation can improve the clinical course of HE and sarcopenia with few side effects. Nonetheless, additional research is necessary to investigate potential adverse impacts of BCAAs, particularly concerning the detrimental effects on cataplerosis in muscles and the generation of ammonia from glutamine in visceral tissues. BCAAs administration alone has been shown to improve HE clinical manifestation, therefore improving quality of life and reducing HE recurrence [68]. However, the studies conducted so far have not shown any significant effect on mortality [57]. It is worth mentioning that malnutrition has been demonstrated to be associated with mortality, the development of complications, reduced survival, and reduced HRQoL in individuals with ACLD [7,8,9]. This highlights the urgent need for an effective and safe treatment for sarcopenia in patients with cirrhosis [20,24]. In patients with sarcopenia, BCAA supplementation has a positive effect on muscle mass, muscle strength, and albumin levels, which can lead to improved survival. The beneficial effects of BCAAs are amplified when they are used in combination with physical exercise and nutritional intervention [84].

This evidence would potentially support the use of BCAA supplements in clinical practice in combination with standard treatments, especially in the subgroups of patients with concomitant HE and sarcopenia. There is a need to identify patients at high risk of malnutrition and sarcopenia who could have an increased benefit from early nutritional intervention and BCAA supplementation. Oral administration seems to be more effective than intravenous administration and should be preferred [61]. Early discontinuation of BCAA administration is associated with reduced benefit, so long-term supplementation should be preferred. A minimum dose of 12 g/d of oral BCAAs is more effective than lower doses, but further studies are needed to evaluate the most adequate dose and duration of BCAA treatment [61].

In vitro, animal, and human studies have also suggested that BCAAs can affect the immunological response and inhibit the proliferation of HepG2 liver tumor cells, in conjunction with decreased insulin-mediated proliferation and reduced expression of vascular endothelial growth factor [62]. These phenomena could contribute to the beneficial effect of BCAAs on the primary and/or secondary prevention of hepatocellular carcinoma. As mentioned previously, impaired nutritional status and sarcopenia are known to be associated with a poor prognosis in cirrhotic patients [63]. Therefore, it is plausible to hypothesize that the beneficial effects of BCAA supplementation observed in patients with HCC may be mediated through improved nutritional status, potentially due to enhanced liver function. However, more research is needed to confirm these findings.

Overall, the use of BCAAs in advanced chronic liver disease is a safe treatment that can have positive effects on the clinical course of HE and sarcopenia and may also improve survival. Further studies (ideally multicenter randomized clinical trials) are needed to confirm these findings and optimize the use of BCAAs in this patient population.
